# Clinical analysis of successful insertion of orthodontic mini-implants in infrazygomatic crest

**DOI:** 10.1186/s12903-023-03081-0

**Published:** 2023-06-01

**Authors:** Yinxue He, Jinan Liu, Rui Huang, Xing Chen, Xueting Jia, Na Zeng, Xiaochuan Fan, Xiaofeng Huang

**Affiliations:** 1 Department of Stomatology and Immunology Research Center for Oral and Systemic Health, Beijing Friendship Hospital, Capital Medical University, No. 95 Yong’an Road, Xicheng District, 100050 Beijing, PR China; 2 Immunology Research Center for Oral and Systemic Health, Beijing Friendship Hospital, Capital Medical University, Beijing, China; 3 National Institute on Drug Dependence and Beijing Key Laboratory of Drug Dependence, Peking University, Beijing, PR China; 4 School of Public Health, Peking University, Beijing, PR China

**Keywords:** Mini-implant, Infrazygomatic crest, Orthodontic

## Abstract

**Background:**

The insertion positions of mini-implant in infrazygomatic crest has been reported, but due to the anatomical variation, the precise location of this site is not clear yet. This study used cone-beam computed tomography (CBCT) to analyze the position and angle of mini-implants successfully inserted in the infrazygomatic crest, with the goal of providing reference data for clinical practice.

**Methods:**

CBCT was used to image 40 mini-implants and their surrounding tissues in adult orthodontic patients who successfully underwent mini-implant insertion in the infrazygomatic crest. The insertion positions and angles of mini-implants were measured, and the thicknesses of buccal and palatal bone adjacent to the mini-implants were also recorded. Then, we proposed the position and implantation angle for infrazygomatic crest insertion. According to the position and angle, the cortical bone thickness and distance to the root of another 54 randomly selected infrazygomatic crests were recorded to verify its feasibility.

**Results:**

In the coordinate system, the implantation position of the 40 successful mini-implants was (-0.4 ± 2, 8.2 ± 2.5) and the implantation angle between the long axis of the mini-implant and horizontal reference plane was 56.4° ± 7.7°. The bone thicknesses on buccal and palatal sides of infrazygomatic crest adjacent to mini-implants were 4.1 ± 2.5 mm and 7.2 ± 3.2 mm, respectively, and the cortical bone thickness was 2.4 ± 0.6 mm. Among 54 infrazygomatic crests, 75.9% of them met the safety and stability requirements. When the implantation height was increased by 1, 2, and 3 mm, the proportions of implants that met requirements for success were 81.5%, 90.7%, and 94.4%, respectively. But, the proportions of eligible implants were limited at implantation angle increases of 5° and 10°.

**Conclusions:**

Using the long axis of the maxillary first permanent molar (U6) as the vertical reference line, mini-implants could be safely inserted in the infrazygomatic crest at a distal distance of 0.4 mm and height of 8.2 mm from the central cementum-enamel junction of U6, with an implantation angle of 56.4°. The success rate increased when the implant height increased, but the proportion of eligible implantation was limited with the increase of implantation angle.

## Background

In recent 20 years, mini-implants have been widely used in orthodontic clinic because of their convenience and strong support. Compared with conventional orthodontic anchorage approaches, mini-implants have the advantages of “absolute” anchorage, high biocompatibility, small size, increased patient comfort, immediate loading, and low cost [1, 2]. Thus, mini-implants are useful in efforts to expand the scope of orthodontic treatment.

The effectiveness of orthodontic treatment largely depends on continuous and effective stabilization of anchorage. Compared with the anchorage of mandibular teeth, the anchorage of maxillary posterior teeth is relatively weak. Therefore, close attention is needed when attempting to strengthen the anchorage of maxillary posterior teeth. The infrazygomatic crest and the alveolar bone between tooth roots are commonly used as mini-implant anchorage positions in the maxillary posterior region. In contrast to interradicular alveolar bone, the infrazygomatic crest has larger bone mass and higher bone density, along with a thicker buccal cortex. The increased cortical bone thickness on the buccal side and at the base of the maxillary sinus provides good support for implants, thereby improving initial stability and increasing the insertion success [3]. The infrazygomatic crest allows a greater range of implant locations, compared with the alveolar bone between tooth roots. However, there are various opinions regarding the optimal site and angle for mini-implants in the infrazygomatic crest. Liou et al. [4] suggested that insertion 14–16 mm above the maxillary occlusal plane could avoid damage to the mesiobuccal root of the maxillary first permanent molar (U6) and the risk of irritation to the buccal mucosa. Considering the thickness of the infrazygomatic crest, Murugesan et al. [5] reported that the optimal position was 12–17 mm above the occlusal plane. They suggested an angle of 55°–70° relative to the maxillary occlusal plane. However, their recommended ranges of implant height and implantation angle were excessively large, hindering usage in clinical practice. Moreover, their measurements were conducted using cone-beam computed tomography (CBCT) data for healthy individuals, rather than data from patients who required implants or with implants.

In this study, we used CBCT images to investigate the successful implantation of mini-implants in the infrazygomatic crest area to conducted an in-depth analysis of the optimal position and implantation angle. Furthermore, we randomly selected another 54 CBCT images of the infrazygomatic crest to validate the application of this implant position and angle, supporting their usage for reference in clinical practice.

## Methods

This study protocol was approved by the Medical Ethics Committee of Beijing Friendship Hospital, Capital Medical University (2021-P2-373–01).

### Clinical data

Samples were collected from adult orthodontic patients who attended the Department of Stomatology of Beijing Friendship Hospital, Capital Medical University from January 2019 to June 2022. The sample size of 40 was calculated based on an alpha of 0.05 and the power of 90%, using a two-sided one-sample* t*-test in PASS version 15.0 (NCSS, LLC. Kaysville, Utah, USA) [6].

Forty implant sites were collected from twenty-one patients (19 on the left, 21 on the right; 4 males and 17 females). The following inclusion criteria were used: adult orthodontic patients with increased maxillary anchorage who underwent mini-implant insertion in the infrazygomatic crest; CBCT had been performed immediately after mini-implant insertion; CBCT images were clear without artifacts; there was no damage to the maxillary sinus, root, or adjacent anatomical structures after implantation; no mini-implant loosening or shedding occurred at the end of treatment; crowns and roots of U6 were intact, and no crown restoration was required. The following exclusion criteria were used: missing teeth (except third molars), supernumerary teeth, severe crowding; craniofacial developmental deformity, maxillofacial surgery, and/or trauma, which resulted in bilateral asymmetry; severe periodontal disease, such that bilateral maxillary posterior teeth clearly exhibit alveolar bone resorption; prior history of orthodontic treatment.

### Image reconstruction

All images were acquired using the same CBCT machine (NewTom 5G Version FP, QR S.r.l, Italy). The following conditions were used for image acquisition: voltage, 110 kV; current, 5 mA; scanning time, 3.6 s; and scanning range, 18 cm × 16 cm. The patient’s head was oriented in a horizontal supine position, and data were transmitted to the computer after image acquisition. CBCT data for 40 mini-implants and surrounding tissues were imported into Dolphin Imaging & Management Solutions software (USA) in the Digital Imaging and Communication in Medicine (DICOM 3.0) file format. Image reconstruction was performed by a single observer on a computer screen with a resolution of 1280 × 1024 under indoor light. The median sagittal plane was regarded as the plane that equalized the patient’s left and right craniofacial regions when the distance between the observer and the display screen was approximately 30 cm. Brightness and grayscale values were adjusted to obtain clear CBCT images.

### Measurement items

#### Implantation coordinates

**Fig. 1:**
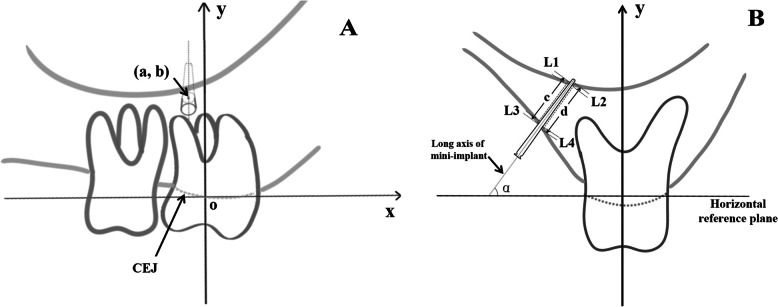
Measurement items. **A** Mini-implant coordinates(a, b): the long axis of the mini-implant (i.e., the line from the center point of the implant crown to the implant tip) which in contact with the buccal surface of the infrazygomatic crest. **B** Implantation angle and bone thickness around the mini-implant: α: Implantation angle formed with the horizontal reference plane; c: Buccal bone thickness d: Palatal bone thickness; (L1 + L2)/2 + (L3 + L4)/2: Cumulative cortical bone thickness

On the sagittal plane, the long axis of U6 (i.e., the line from the central fossa to the root bifurcation) was regarded as the y-axis. The origin was regarded as the location where the central cementum-enamel junction (CEJ) of U6 was projected onto the y-axis. A reference line perpendicular to the y-axis, passing through the origin and parallel to the median sagittal plane, was regarded as the x-axis. The mesial direction of U6 was the positive direction on the x-axis (Fig. 1A). Implantation coordinates (a, b) were recorded at the point where the long axis of the mini-implant (i.e., the line from the center point of the implant crown to the implant tip) was in contact with the buccal surface of the infrazygomatic crest.

#### Implantation angle

The horizontal reference plane of U6 was perpendicular to the long axis of U6. The Implantation angle, regarded as α, between the long axis of mini-implant and the horizontal reference plane was measured (Fig. 1B).

#### Thicknesses of buccal and palatal bone around mini-implants

Based on continuous observations, the coronal plane through the tip of the mini-implant was selected as the plane for measurement of the thicknesses of buccal and palatal bone, as well as the thickness of cortical bone. All measurements were performed by the same clinician at 2-week intervals to ensure consistency.

Buccal (or palatal) bone thickness: Measurement began where the buccal (or palatal) side of the mini-implant penetrated cortical bone. It ended where the long axis parallel line of the mini-implant penetrated cortical bone within the maxillary sinus floor. The distance between those two points was regarded as buccal (or palatal) bone thickness (Fig. 1B).

Cortical bone thickness: The first measurement began where the mini-implant penetrated cortical bone on the buccal (or palatal) side; it ended where the mini-implant penetrated cancellous bone through lines parallel to the long axis of the mini-implant at the starting point. The second measurement began where the upper extension line penetrated cancellous bone into cortical bone near the maxillary sinus floor; it ended where the upper extension line entered the maxillary sinus and penetrated the cortex near the maxillary sinus floor. The mean value of the sum of the two measurements between buccal and palatal sides was the thickness of cortical bone at this site (Fig. 1B).

#### Inspection site and angle

Based on the predicted position and angle in cases of successful implantation, the sample size was also calculated by PASS software using an α value of 0.05 [6]. 54 infrazygomatic crests in 27 patients were selected to simulate the insertion of mini-implants in a consistent manner, and the thickness of cortical bone was measured with respect to the long axis of the implants. Considering an implant diameter of 2.0 mm, we investigated root visibility at 1.0 mm from the long axis of each mini-implant.

The following inclusion criteria were used: patients with CBCT images of the entire skull; no obvious crowding among maxillary posterior teeth; intact crowns and roots of U6, without restorations or severe abrasions. The exclusion criteria were used as described above.

### Statistical methods

Data were analyzed using SPSS 19.0 statistical software. The Kolmogorov–Smirnov test was used to evaluate the normality of the data. In Tables 1 and 2, bone thickness was expressed as median (P_25_, P_75_) and mean ± standard deviation. The incidences of mini-implant success and was presented as the percentages of the number of related sites divided by the total number of sites. Differences were assessed using *t*-tests, and *P*-values < 0.05 were considered statistically significant.

**Table 1:** CBCT analysis of 40 infrazygomatic crest mini-implants and adjacent bone tissues in 21 patients

	$\overline{x}\pm \mathrm{S }$	Median (P_25-_P_75_)	min	max
a	-0.4 ± 2.8	0 (-1.4,1.1)	-7.5	2.3
b	8.2 ± 2.5	8.3 (7,9.4)	3.1	14.4
α (°)	56.4 ± 7.7	55.9 (50.3,62)	43.7	73.1
Thickness of the buccal side bone (mm)	4.1 ± 2.5	3.5 (2.6,4.9)	0.9	13.8
Thickness of the palate side bone (mm)	7.2 ± 3.2	6.7 (5,9.2)	1.8	19.7
Cortical bone thickness (mm)	2.4 ± 0.6	2.3 (1.9,2.9)	1.5	3.5

**Table 2:** Cortical bone thickness (mm) of infrazygomatic crest mini-implants at (-0.4, 8.2) with angle of 56.4°

	$\overline{x}\pm \mathrm{s }$	Median (P_25-_P_75_)	min	max
Sum of cortical bone thickness	2.6 ± 0.8	2.5 (2.1,3.2)	1.1	4.5

## Results

There was no significant difference between the two measurement results (*P* > 0.05). The intraclass correlation coefficient was 0.972, indicating that measurements were reproducible.

The 40 mini-implants successfully inserted in the infrazygomatic crest had an implant position of (-0.4 ± 2 mm, 8.2 ± 2.5 mm) and an implantation angle of 56.4° ± 7.7°. The maximum horizontal and vertical differences in implant position were 9.8 mm and 11.3 mm, respectively (Table 1). We suspect that these differences were influenced by variations in infrazygomatic crest morphology.

Palatal bone thickness adjacent to the mini-implants (median, 6.7 mm; mean, 7.2 mm) was significantly greater than buccal bone thickness (median, 3.5 mm; mean, 4.1 mm) (*P* < 0.05). The average and the median of cortical bone thickness around the 40 mini-implants was 2.4 mm and 2.3 mm, respectively. The minimum thickness was 1.5 mm. There were no statistically significant differences between left and right sides or male and female patients (*P* > 0.05, Table 2).

**Fig. 2:**
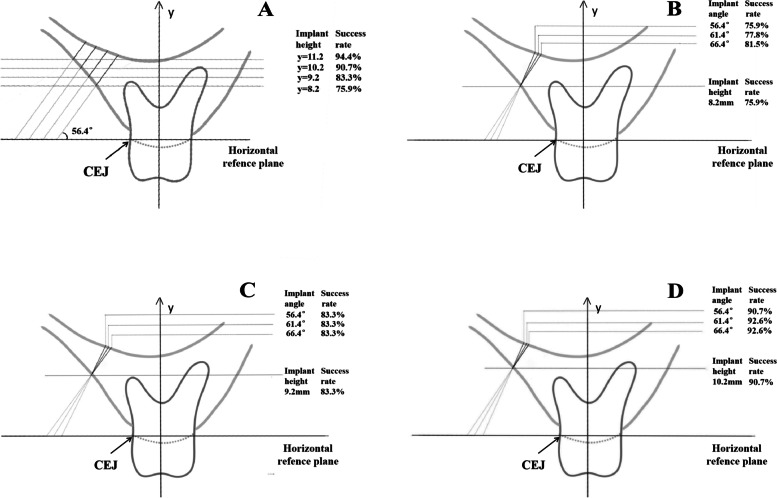
Influence of different implantation sites and angles on the success rate. **A** The success rates when the implantation height was changed at infrazygomatic crest with angle of 56.4°. **B-D** The success rates when the implantation angle was changed at the 8.2 mm, 9.2 mm and 10.2 mm heights of infrazygomatic crest

Commonly used mini-implants have a diameter of 1.2–2 mm. According to the criterion of 2-mm-diameter mini-implants, 54 CBCT images of the infrazygomatic crest were randomly selected. We regarded the distance from long axis of each mini-implant to tooth root at least 1.0 mm and total cortical bone thickness of > 1 mm as the successful implantation. The mini-implant long axis was simulated at 56.4° with U6 long axis at coordinates (-0.4, 8.2) in the same reference manner. Among the 54 cases, average cortical bone thickness around hypothetical mini-implant was 2.6 mm and the median thickness was 2.5 mm (Table 2). All of them had a total cortical bone thickness of > 1 mm which the minimum thickness was 1.1 mm. In 41 cases (75.9%), the mini-implant did not touch the dental root at the long axis of the implant, nor at 1 mm in the proximal and distal directions from the implant (Table 3). If the implant height was increased by 1 mm, 2 mm, and 3 mm (Fig. 2A), the proportion without contact increased to 83.3%, 90.7%, and 94.4%, respectively (Table 4). At implant heights of 8.2 mm, 9.2 mm, and 10.2 mm, increases of 5° and 10° in the implantation angle (Fig. 2B, C, D) led to limited increases in the proportions of eligible implants: 1.9% and 3.7% for 5° and 10°, respectively, at 8.2 mm, and 1.9% for 5° at 10.2 mm. No other changes in implantation angle met the requirements for successful implantation (Table 5).

**Table 3:** Root contact among 54 infrazygomatic crest mini-implants at (-0.4, 8.2) with angle of 56.4°

	No contact was found at 1 mm proximal the mini-implant long axis	No contact at the long axis of the mini-implant	No contact was found at 1 mm distal the mini-implant long axis	All no contact
Number of cases	49	50	47	41
Proportion	90.7%	92.6%	87.0%	75.9%

**Table 4:** Proportions of mini-implants 1 mm above root at (-0.4, Y) with angle of 56.4°

	y = 8.2	8.2 < y ≤ 9.2	9.2 < y ≤ 10.2	10.2 < y ≤ 11.2	11.2 < y still unable to meet the requirements
Number of cases	41	4	4	2	3
Proportion	75.9%	5.6%	9.3%	3.7%	5.6%
Cumulative proportion	75.9%	83.3%	90.7%	94.4%	-

**Table 5:** Proportions of 54 infrazygomatic crest mini-implants with sufficient angle to reach > 1 mm from the root during (-0.4, y) implantation

Height	y = 8.2			y = 9.2			y = 10.2		
Angle	α ≤ 56.4°	56.4° < α ≤ 61.4°	61.4° < α ≤ 66.4°	α ≤ 56.4°	56.4° < α ≤ 61.4°	61.4° < α ≤ 66.4°	α ≤ 56.4°	56.4° < α ≤ 61.4°	61.4° < α ≤ 66.4°
Number of cases	41	1	2	4	0	0	4	1	0
Proporti-on	75.9%	1.9%	3.7%	7.4%	0%	0%	7.4%	1.9%	0%
Cumulat-ive proporti-on	75.9%	77.8%	81.5%	83.3%	83.3%	83.3%	90.7%	92.6%	92.6%

## Discussion

The infrazygomatic crest is a common site for insertion of mini-implants in the maxillary posterior region. Mini-implants in this region are higher than the interradicular site between posterior teeth, enabling closer proximity to the center of maxillary impedance. The stronger the overall effect on maxillary dentition is, the better the effect of tooth distal movement is enhanced [4].

In study of the implant position, the establishment of reference plane is very important for measurement. Liou [4] used the maxillary occlusal plane as the reference plane. However, the occlusal plane is an artificial plane formed from the proximal point of the bilateral maxillary central incisors to the mesiobuccal cusps apex of U6. The edge of the incisor and the cusps of the molar are usually worn in adult, so the occlusal plane is relatively unstable in different ages and individuals. In another study, the alveolar bone crest has been used as a reference point for measurement [7]. But, the alveolar bone crest is a generally unstable reference point which can be influenced by periodontal inflammation. Santos et al. [8] used the Frankfort horizontal plane as a reference plane, however the plane is not visual enough for application in clinic. In the present study, we selected the long axis of U6 and its buccal central of CEJ as the reference. The infrazygomatic crest is a bone ridge that passes through the buccal side of U6, the long axis of U6 body appears a more intuitive during implantation. The buccal central point of U6 CEJ is a stable position and easy to be detected in clinic.

Previous studies explored the range of appropriate heights and angles for mini-implant insertion in the infrazygomatic crest. Most of those studies used bone thickness as the reference standard. Baumgaertel et al. [9] used the buccal CEJ of U6 as a reference point to measure bone thickness at the infrazygomatic crest, and greatest bone thickness was found at 11.48 mm above CEJ. Santos et al. [8] reported the thicknesses of 2.49 mm and 2.29 mm at 2 mm and 4 mm above the distal buccal root apex of U6, respectively. Vargas found that in the vertical direction, the corresponding position of the mesiobuccal root of U6 was an optimal location [10]. These studies only measured bone thickness at the infrazygomatic crest without considering implantation angle. Liou et al. [4] found that the full thickness of the infrazygomatic crest varied by 5.2–8.8 mm as the implantation angle varied from 40° to 75° based on the maxillary occlusal plane. Further, Du et al. comprehensively investigated bone conditions around mini-implants and explored the relationship between the implant and the tooth root. They reported that if an implant was between U6 and the second permanent molar (U5), a height of 15 mm above the posterior tooth occlusion plane was appropriate with enough bone volume, along with an implantation angle of 60°–70° [11].

Compared with the thicknesses of bone on the buccal and palatal sides, the anatomical conditions of cortical bone have more effects on the initial stability and long-term success of mini-implants. According to Motoyoshi et al. [12], mini-implants required ≥ 1 mm of cortical bone to maintain stability.

In the present study, we first selected the images from cases of successful implantation at the infrazygomatic crest to observed the actual positions and angles of mini-implants, which is more reliable. Based on a vertical reference line from the long axis of U6, we found that the position of implants successfully anchored was 8.2 mm height and 0.4 mm distal to the central CEJ with the implantation angle of 56.4° relative to the horizontal reference plane. The bone thickness of the infrazygomatic crest of the buccal and palatal side adjacent to the mini-implant was 3.5 mm and 6.7 mm respectively, the average thickness of bilayer cortical bone in the peri-implant crown-root orientation (i.e., the sum of cortical bone thicknesses at the infrazygomatic crest and maxillary sinus floor) was 2.4 mm, which exceeded the standard for successful insertion of mini-implant and indicates that this site can provide sufficient bone mass to support mini-implants.

In previous experiments, researchers have generally performed measurements without exploring relevant applications. To demonstrate the feasibility of applying our findings to clinical practice, 54 CBCT images of infrazygomatic crest were seleted. Using the position and the implantation angle of 56.4° we proposed, there was a 75.9% probability of meeting the requirements for cortical bone thickness and distance from the root. Subsequently, we varied the implant height and implantation angle. When the implantation angle was 56.4°, the success rate increased as implant height increased. If the implant height was more than 10.2 mm, the proportion without contact increased to more than 90% (Fig. 2A, Table 4), which provided a good implantation condition for clinical application. Although bone thickness at the infrazygomatic crest gradually decreased with the increase of insertion height, the total cortical bone thickness at these sites were still enough for implantation stability. However, when we changed the implantation angle while maintaining a specific height, it is found that the success rate exhibited minimal variation, suggesting that specific ranges of variation in implantation angle have limited effects on the success of mini-implant insertion. Therefore, when considering the implantation in the infrazygomatic crest, if the position and the implantation angle of 56.4°we prepared are not suitable for implantation, the height of the implant site should be considered first, but not implantation angle.

The position of maxillary sinus is another important interference factor of implanting anchorage in the infrazygomatic crest. Our previous findings suggested that mini-implants in the infrazygomatic crest can penetrate the thick cortical bone plate [13]. Therefore, in order to maintain the initial stability and health of the maxillary sinus, the length of the mini-implant should be carefully selected, and the implant depth should also be considered at a higher insertion position.

## Conclusions

Taken together, based on CBCT images of successful insertion of mini-implants at the infrazygomatic crest, we drew the following conclusion: Using the long axis of the maxillary first permanent molar as the vertical reference line, a point 0.4 mm distal to and 8.2 mm above the CEJ on the buccal side of U6 is appropriate for mini-implant insertion in the infrazygomatic crest and an implantation angle of 56.4° relative to the long axis of U6 is safe. The success rate increases with increasing implant height. But the proportion of eligible implants was limited, when the implantation angle increased.

## Data Availability

All data generated or analysed during this study are included in this published article.

## Abbreviations

CBCT: Cone-beam computed tomography
U6: Maxillary first permanent molar
CEJ: Cementum-enamel junction
U5: Maxillary second permanent molar
